# The relationship between childhood maltreatment and trauma and psychosis is not moderated by parental mental health

**DOI:** 10.1186/s12888-025-07190-8

**Published:** 2025-08-06

**Authors:** Nina Mørkved, Pia Sophie Bryntesen, Ida Marie Eggen, Erik Johnsen, Rune Andreas Kroken, Christoffer Andreas Bartz-Johannessen, Åshild Huiberts, Camilla Burgess, Inge Joa, Maria Rettenbacher, Else-Marie Løberg

**Affiliations:** 1 Department of Research and Innovation, Helgeland Hospital, Sandnessjøen, Norway; 2 Institute of Psychology, The Arctic University of Norway, Tromsø, Norway; 3 Faculty of Psychology, University of Bergen, Bergen, Norway; 4 Division of Psychiatry, Haukeland University Hospital, Bergen, Norway; 5 Department of Clinical Medicine, University of Bergen, Bergen, Norway; 6 Stavanger University Hospital, Stavanger, Norway; 7 Department of Psychiatry and Psychotherapy, Medical University Innsbruck, Innsbruck, Austria; 8 Institute of Public Health, Faculty of Health Sciences, University of Stavanger, Stavanger, Norway

**Keywords:** Schizophrenia, Psychosis, Psychotic symptoms, Childhood trauma, Adverse events, Parental mental health

## Abstract

**Background:**

Childhood maltreatment and trauma (CMT) increase the risk for schizophrenia spectrum disorders (SSDs) and the severity of psychosis symptoms. Few studies have considered the possible influence of parental mental health on the relationship between CMT and symptoms of psychosis. Possibly, parental mental health problems (MHP) confound this relationship by increasing both the genetic vulnerability for psychosis and the potential for sub-optimal childhood environments. The aim was to examine the potential influence of parental MHP on the relationship between CMT and symptoms of psychosis. We hypothesized a positive and dose-dependent association between overall CMT and symptoms of psychosis not moderated by parental MHP.

**Methods:**

Patients with SSDs (*N* = 133) from the Bergen-Stavanger-Innsbruck-Trondheim (BeStInTro) study were included and assessed for CMT by the Childhood Trauma Questionnaire - Short Form, psychosis symptoms by The Positive and Negative Syndrome Scale and parental mental health by means of focused patient interviews.

**Results:**

Regression analyses showed a dose-response relationship between CMT and overall psychosis symptom severity and negative symptom severity, further supported by t-tests showing that SSD patients with CMT showed more psychosis symptoms compared to SSD patients with no CMT. Multiple regression analysis with interaction term showed that the association between CMT and psychosis symptom severity was independent, and not moderated, by parental MHP.

**Conclusion:**

A dose-dependent relationship between CMT and psychosis symptoms emerged, not moderated by parental MHP, suggesting that CMT has an independent and true effect on psychosis symptoms.

**Supplementary Information:**

The online version contains supplementary material available at 10.1186/s12888-025-07190-8.

## Introduction

Childhood maltreatment and trauma (CMT) increase the risk for psychosis and schizophrenia spectrum disorders (SSDs; [1–5]). Typically, these experiences are conceptualized as childhood trauma due to their potentially trauma inducing quality and includes adverse childhood experiences such as physical abuse and neglect, emotional abuse and neglect, and sexual abuse [6]. CMT usually takes place in the context of a relationship of trust or power, such as the parent-child relationship, and CMT often entails harm or threat of harm caused by commission or omission by the caregiver of a child [7, 8]. Kessler, McLaughlin [9] found that CMT and maladaptive family functioning including parental mental health problems (MHP) was linked to a high risk of MHP in the offspring. Parental factors including MHP may increase the risk of CMT [10–12]. Manning and Gregoire suggest that parental factors may directly impact their offspring via shared genetics, intrauterine environment and exposure to the parental mental illness itself, or indirectly via e.g. socioeconomic disadvantage and MHP induced marital conflict [12]. Since there is a lack of a clear understanding of the mechanisms for the CMT – psychosis relationship [4], it is imperative for the prevention of psychosis to gain knowledge on whether this relationship is of a true or independent nature, or whether CMT is more a marker of a genetic vulnerability through parents with MHP [13, 14]. A systematic review and meta-analyses found that the causal relationship between CMT and mental health was moderated by pre-existing factors, suggesting that e.g. socioeconomic disadvantages and genetic liability may influence this relationship [15]. The risk ratio and lifetime risk of mental disorder are highly increased in offspring of parents with MHP [16, 17]. Parental mental health may confound the CMT and psychosis relationship by creating both genetic vulnerability for psychosis and sub-optimal childhood environments.

Understanding the mechanisms underlying the impact of CMT on psychosis is of etiological and clinical importance. Varese, Smeets [3] suggested that preventing CMT could reduce SSDs by about 33% if all other risk factors were held constant and assuming causality. Further, CMT may worsen the prognosis of SSDs and influence treatment response [18–21]. Moreover, a dose-response relationship between CMT and severity of psychosis symptoms in SSDs have consistently been shown [22–24]. Few studies have however examined the association of CMT and psychosis symptom severity in SSDs while also considering the possible moderating influence of parental MHP.

The aim of the present study was to examine the moderating effect of parental MHP on the relationship between CMT and symptoms of psychosis in SSDs. It was hypothesized that there would be a dose dependent positive association between CMT severity and psychosis symptom severity not moderated by parental MHP.

## Materials and methods

### Sample

The current study was based on cross-sectional data from the Bergen-Stavanger-Innsbruck-Trondheim (BeSt InTro) study; a rater-blind, randomized, controlled pragmatic trial comparing the effectiveness and safety of amisulpride, aripiprazole, and olanzapine in Bergen, Trondheim and Stavanger, Norway, and Innsbruck, Austria, see Johnsen et al. [25] for details and inclusion and exclusion criteria. The present study included 133 patients 18 years or older, diagnosed with SSDs (ICD-10 diagnoses F20-29) [26] by means of the Structured Clinical Interview for DSM-IV axis 1 disorders [27] by trained physicians and psychologists. See Table 1 for details on clinical and demographic characteristics.

**Table 1:** Mean (SD) or n (%) for clinical and demographic characteristics by CMT and no CMT group

Baseline characteristics	No CMT group (*n* = 65)^a^	CMT group (*n* = 68)^a^	Statistics (t or Χ^2^)^b^	*p*	Total (*N* = 133)
Age, years	30 (12.6)	30.1 (11.9)	− 0.021	0.983	30.1 (12.2)
Male	43 (66.2%)	40 (58.8%)	0.481	0.488	83 (62.4%)
Caucasian	54 (90%)	55 (88.7%)	0	1	109 (89.3%)
Years of education	12.6 (3.2)	11.8 (2.7)	1.654	0.101	12.2 (3)
Living alone (yes)	21 (34.4%)	30 (47.6%)	1.716	0.190	51 (41.1%)
Employed (yes)	14 (23%)	14 (22.2%)	0	1	28 (22.6%)
DDD^c^	1.1 (0.5)	1.1 (0.4)	0.145	0.885	1.1 (0.5)
DUP, weeks	43.4 (72.5)	68 (118.1)	− 1.168	0.247	56 (98.7)
Psychosis onset age, years	23.3 (7.4)	24.8 (9.9)	− 0.845	0.400	24.1 (8.8)
Diagnosis					
Schizophrenia	23 (35.4%)	38 (55.9%)	4.828	0.028*	61 (45.9%)
Schizotypal disorder	0 (0%)	2 (2.9%)	0.463	0.496	2 (1.5%)
Delusional disorder	8 (12.3%)	7 (10.3%)	0.009	0.926	15 (11.3%)
Brief psychotic disorder	10 (15.4%)	7 (10.3%)	0.383	0.536	17 (12.8%)
Schizoaffective disorder	6 (9.2%)	2 (2.9%)	1.346	0.246	8 (6%)
Other psychotic disorder	1 (1.5%)	0 (0%)	0.001	0.982	1 (0.8%)
Unspecified psychotic disorder	5 (7.7%)	5 (7.4%)	0	1	10 (7.5%)
Smoking ^d^ (yes)	33 (54.1%)	45 (73.8%)	4.301	0.038*	78 (63.9%)
CAUS (abuse or dependence)	4 (6.6%)	7 (10.8%)	0.272	0.602	11 (8.7%)
CDUS (abuse or dependence)	17 (27.9%)	17 (26.2%)	0	0.987	34 (27%)
Antipsychotic naive	22 (33.8%)	18 (26.5%)	0.545	0.460	40 (30.1%)
PANSS total	70.6 (19)	77.2 (15.2)	− 2.209	0.029*	74 (17.4)
PANSS positive	18.5 (5.7)	20.7 (5.5)	− 2.271	0.025*	19.6 (5.7)
PANSS negative	15.9 (6.1)	17.9 (5.9)	− 1.987	0.049*	16.9 (6.1)
PANSS general psychopathology	36.3 (10.2)	38.6 (7.9)	− 1.453	0.149	37.5 (9.1)
CGI	4.7 (1.1)	4.9 (0.9)	− 1.562	0.121	4.8 (1)
GAF	39.1 (9.6)	37.9 (11)	0.677	0.500	38.5 (10.4)
CDSS	5.8 (4.8)	8.6 (5.3)	− 3.139	0.002**	7.2 (5.2)
BMI	24.5 (4.2)	25.4 (5.7)	− 1.012	0.314	24.9 (5)
Mental disorder, mother	12 (18.5%)	12 (17.6%)	0	1	24 (18%)
Bipolar	4 (6.2%)	4 (5.9%)	0	1	8 (6%)
Schizophrenia	0 (0%)	3 (4.4%)	1.274	0.259	3 (2.3%)
Suicide attempts	1 (1.5%)	4 (5.9%)	0.741	0.389	5 (3.8%)
Suicide	0 (0%)	0 (0%)	-	-	0 (0%)
Alcohol use/dependence	1 (1.5%)	6 (8.8%)	2.227	0.136	7 (5.3%)
Substance use/dependence	2 (3.1%)	2 (2.9%)	0	1	4 (3%)
Other	5 (7.7%)	2 (2.9%)	0.703	0.402	7 (5.3%)
Mental disorder, father	9 (13.8%)	11 (16.2%)	0.018	0.894	20 (15%)
Bipolar	4 (6.2%)	3 (4.4%)	0.004	0.951	7 (5.3%)
Schizophrenia	2 (3.1%)	2 (2.9%)	0	1	4 (3%)
Suicide attempts	1 (1.5%)	3 (4.4%)	0.213	0.644	4 (3%)
Suicide	1 (1.5%)	1 (1.5%)	0	1	2 (1.5%)
Alcohol use/dependence	7 (10.8%)	14 (20.6%)	1.728	0.189	21 (15.8%)
Substance use/dependence	2 (3.1%)	3 (4.4%)	0	1	5 (3.8%)
Other	1 (1.5%)	2 (2.9%)	0	1	3 (2.3%)
CTQ-SF sum	31.9 (4.3)	54.2 (15.2)	− 11.634	< 0.001***	43.3 (15.9)
Emotional abuse	7 (2)	12.9 (5.1)	− 8.969	< 0.001***	10 (4.9)
Physical abuse	5.3 (0.7)	8.5 (4.1)	− 6.251	< 0.001***	6.9 (3.4)
Sexual abuse	5 (0.2)	7.8 (4.6)	− 4.879	< 0.001***	6.4 (3.6)
Emotional neglect	8.3 (2.6)	15.2 (5.1)	− 9.756	< 0.001***	11.8 (5.3)
Physical neglect	6.3 (1.5)	9.9 (3.8)	− 7.377	< 0.001***	8.1 (3.4)

*p <.05. **p <.01. ***p <.001. *p* = *p*-value; *n* = Number of individuals in subsample; *N* = Number of individuals in total sample; SD = Standard deviation; *t* = *t*-value; X^2^ = Chi-square test statistic; CMT = Childhood maltreatment and trauma; DDD = Defined daily dose of antipsychotic medication; DUP = Duration of Untreated Psychosis; CAUS = Clinical Alcohol Use Scale; CDUS = Clinical Drug Use Scale; PANSS = Positive and Negative Syndrome Scale; CGI = Clinical Global Impression Scale; GAF = Global Assessment of Functioning; CDSS = Calgary Depression Scale for Schizophrenia; BMI = Body Mass Index; CTQ-SF = Childhood Trauma Questionnaire Short-Form. ^a^SD for continuous variables and % for categorical variables. ^b^Chi-square tests for % and *t*-tests for SD. ^c^DDD-values from the entirety of the BeSt InTro study, not baseline. ^d^Nicotine cigarettes

### Assessments

#### Childhood maltreatment and trauma (CMT)

CMT was assessed with the Norwegian version [28] of the 28 item self-report questionnaire the Childhood Trauma Questionnaire – Short Form [CTQ-SF; 6]. The CTQ-SF consists of five subscales that measure five subtypes of CMT: physical, sexual and emotional abuse, and emotional and physical neglect. The items are rated on a 5-point Likert scale ranging from one (never), two (rarely), three (sometimes), four (often), to five (very often). The CTQ-SF has shown good specificity, sensitivity, internal consistency and test-retest reliability as well as good to excellent reliability for the subscales and the total scale [6, 29]. The present study was based on CTQ-SF scores obtained six weeks after study inclusion, increasing the chance of the patients to be in a stable clinical phase, thus increasing validity. The overall reliability estimates for the CTQ-SF in the present study were strong with an overall Cronbach’s alpha = 0.866. The CTQ-SF scores were categorized into none, low, moderate and severe levels according to the threshold scores from the CTQ-SF manual [30]. Further, a dichotomous variable was created by grouping none and low levels as the no CMT group and the moderate to severe levels as the CMT group [30].

#### Parental history of mental health problems

Information on paternal and maternal history (separate and together) of MHP was collected through a patient interview conducted by a trained research nurse at baseline with the following question: “Have any of the patient’s first-degree relatives had any of the following conditions? (based on all available information)”, yielding information on the following parental MHPs: known bipolar disorder, schizophrenia, alcohol use or dependence, substance use or dependence, other specified MHPs specified by the patient during the interview (reported as “anxiety, personality disorder, eating disorder, burn-out, bipolar disorder, “crazy*”*, hospitalization in a psychiatric unit and violent and lacking empathy”), and suicide attempts. Answering no diagnosis and missing data was categorized as parental MHP not present. Answering known or likely parental diagnosis was categorized as parental MHP present, yielding four groups based on having a (1) mother, (2) father, (3) both or 0) neither with a history of MHP.

#### Symptoms of psychosis

Psychosis symptoms were assessed by the Positive and Negative Syndrome Scale (PANSS) by means of the Structural Clinical Interview for the Positive and Negative Syndrome Scale [SCI-PANSS; 30] at baseline. PANSS consists of a total of 30 items split into positive, negative and general psychopathology subscales. Each item is rated on a 7-point Likert scale ranging from 1 to 7. PANSS have shown strong validity, reliability and sensitivity [31, 32], and has been validated in Norway [33].

#### Other mental health and functioning variables

To characterize the patient group also other mental health and functioning variables and history was assessed (see Table 1): defined daily dose of antipsychotic medication; duration of untreated psychosis; Clinical Alcohol/Clinical Drug Use Scale; Clinical Global Impression Scale; Global Assessment of Functioning; Calgary Depression Scale for Schizophrenia (CDSS), Body Mass Index assessed at baseline.

### Statistical analyses

All models were fitted using R version 4.22 [34]. A *p*-level of < 0.05 was considered statistically significant for all analyses. Measures are presented as number (*n*) and percentages (*%*), or as mean (*M*) and standard deviations (*SD*). The adjusted R^2^ (R^2^adj) was used as a measure of the goodness of fit which was assessed as small if < 0.09, moderate between 0.1 and 0.3 and large effect if > 0.3 [35]. Model assumptions underlying linear multiple regression were checked in R, including assumptions of linearity, homoscedasticity (homogeneity of variance), and normality. The residuals were checked for normality using a QQ-plot. The residuals were also checked for multicollinearity, homoscedasticity, that the data met the linear assumption, and if the confounding variables were incorporated in an appropriate manner. All assumptions were adequately met in the present study.

Independent sample *t*-tests (continuous variables) or Chi-square tests (categorical variables) were used to compare the relation between the demographic variables and the CMT and no CMT groups. The Fisher’s exact test was used to verify the *p*-values from the Chi-square tests for the categorical variables with few observations, as an extra quality measure. For the linear regression analyses, all models were fitted to the data using the following dependent variables (1) PANSS total scale, (2) PANSS subscales (positive, negative, general psychopathology). Age, sex, parental MHP, CTQ-SF sum score, as well as the interaction between parental MHP and the CTQ-SF sum score, were included as independent variables. Age and the CTQ-SF sum score were included as continuous variables, whereas sex and parental MHP were included as categorical variables. The CTQ-SF sum score was used as a predictor for the dependent variables, whereas age and sex were included as confounders. The interaction term was included to examine whether parental MHP moderated the overall level of the CTQ-SF sum score.

## Results

### Demographic and clinical data

**Fig. 1:**
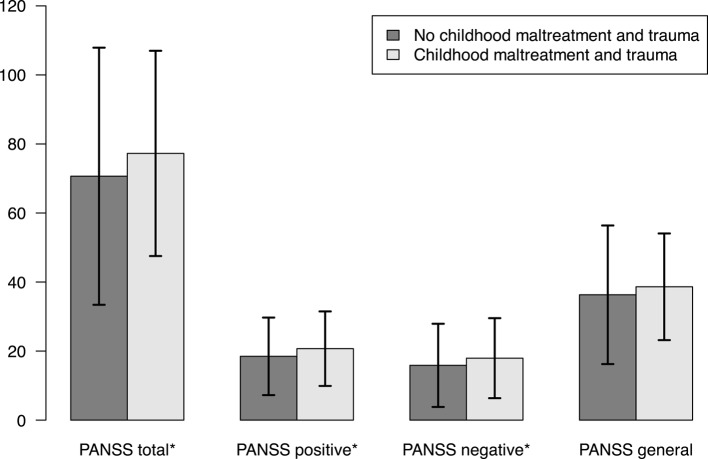
Means PANSS Scores and 95% confidence intervals for PANSS Total and PANSS Positive, Negative and General Psychopathology Subscales by Childhood Maltreatment and Trauma Group. *Difference *p* <.05

See Table 1 for details on clinical and demographic characteristics and statistics by CMT status. A majority, 97 of 133 patients reported no parental MHP, while 36 reported parental MHP, 24 reported maternal MHP and 20 reported paternal MHP (these two groups were overlapping), 16 reported only maternal MHP, 12 reported only paternal MHP and 8 reported both. When comparing the CMT and no CMT groups, few statistically significant group differences for the clinical and demographic variables emerged (see Table 1). There were however more patients diagnosed with schizophrenia as well as higher scores on the CDSS and more nicotine smoking in the CMT group compared to the no CMT group. Further, the CMT group showed higher PANSS total, PANSS positive and PANSS negative subscale scores than the no CMT group (see Fig. 1).

### Parental MHP as moderator for the association between CMT and PANSS total scores

The first multiple regression models examined CTQ-SF sum score and parental MHP as an interaction term on the PANSS total scores, controlling for age and sex (see Table 2).

**Table 2:** Results of the multiple regression analyses of CTQ-SF sum score and parental mental health problems as an interaction term on the PANSS total scale score and subscale scores, controlling for age and sex

	PANSS total scale		PANSS positive subscale		PANSS negative subscale		PANSS general psychopathology subscale	
	Estimate^b^	*p*	Estimate	*p*	Estimate	*p*	Estimate	*p*
Intercept^a^	70.616	0	17.898	0	16.836	0	35.963	0
CMT^c^	0.222	0.049*	0.046	0.213	0.087	0.024*	0.084	0.146
Age	− 0.215	0.091	0.013	0.765	− 0.134	0.002**	− 0.091	0.167
Sex	− 1.081	0.736	− 0.997	0.356	0.308	0.779	− 0.312	0.851
Parental Mental Health Problems								
Maternal	− 2.100	0.877	− 1.171	0.798	0.712	0.878	− 1.782	0.801
Paternal	19.615	0.143	0.285	0.949	8.661	0.059	10.523	0.131
Both	− 44.604	0.141	− 10.199	0.317	− 2.665	0.796	− 31.91	0.044*
CMT X Maternal	0.112	0.709	0.001	0.995	0.015	0.887	0.101	0.519
CMT X Paternal	− 0.252	0.356	− 0.02	0.829	− 0.121	0.198	− 0.107	0.455
CMT X Both	0.852	0.205	0.186	0.412	− 0.023	0.919	0.695	0.048*

*p <.05. **p <.01. *p* = *p* value; *β* = beta, regression coefficient; PANSS = the Positive and Negative Syndrome Scale; CMT = Childhood maltreatment and trauma; CTQ-SF = Childhood Trauma Questionnaire Short-Form. ^a^Mean value of dependent variables when all independent variables equal 0. ^b^Estimate of the expected change in independent variable with one unit change of dependent variable (*β)*. ^c^CTQ-SF sum score

The first model showed that the CTQ-SF sum score was statistically significantly associated with PANSS total scores (*p* =.049), controlling for age and sex (see Table 2). The analysis showed no moderating effect of parental MHP on the PANSS total scale score (see Appendix for details). The association between the CTQ-SF sum score and the PANSS total scale score is indicative of a dose-response relationship, where an increase of 1 on the CTQ-SF sum score would yield an average increase of the PANSS total scale score by 0.22. To illustrate, an increase of 50 in the CTQ-SF total score would yield an average increase of 11 in the PANSS total scale score in this sample. However, statistics indicate poor goodness of fit for the overall model, R^2^adj = 0.033, *F*(9,123) = 1.5, *p* =.154.

### Parental MHP as moderator for the association between CMT and PANSS positive, negative and general psychopathology scores

Linear multiple regression models examined CTQ-SF sum score and parental MHP as an interaction term on the PANSS positive, negative, and general psychopathology subscale scores, controlling for age and sex (see Table 2). The analyses showed no statistically significant association of CTQ-SF sum score and PANSS positive subscale score (*p* =.213), nor did the analyses show any moderation effect of parental MHP (see Appendix for details). The model showed poor model statistics, R^2^adj = − 0.03, *F*(9,123) = 0.54, *p* =.842. The model using the PANSS negative subscale score as outcome was statistically significant, R^2^adj = 0.08, *F*(9,123) = 2.28, *p* =.021. The analyses showed a statistically significant association of the CTQ-SF sum score and the PANSS negative subscale score (*p* =.024). The analyses did not show any moderation effect of parental MHP.

The last model showed no statistically significant association of the CTQ-SF sum score and the PANSS general psychopathology subscale score (see Appendix for details). While the overall model was not significant, R^2^adj = 0.04, F(9,123) = 1.69, *p* =.099, the analyses showed a possible small moderating effect of parental MHP on the relation of the CTQ-SF sum score and the PANSS general psychopathology subscale score (*p* =.048). Patients with MHP in both parents may exhibit a slightly stronger effect of CTQ-SF on PANSS general (see Table 2). However, this estimated moderation effect is small, it is only based on the eight patients having MHP in both parents and should therefore be interpreted carefully until replicated in a larger sample.

## Discussion

The present study examined the moderating effect of parental MHP on the relationship between CMT and psychosis in SSDs. We found an association between CMT and psychosis symptom severity in SSDs, especially for the PANSS total scores and the PANSS negative subscale scores. Parental MHP was not found to moderate the association of CMT and psychosis symptom severity in our sample of SSDs, suggesting that the effect of CMT on psychosis symptom severity is independent of having parental MHP.

Our findings are supported by a recent study showing associations between CMT and adult psychiatric disorders, after controlling for shared genetic and environmental factors in study of 25 000 twins [36]. The current results are further supported by previous studies emphasizing CMT as a risk factor for psychosis and SSDs [1, 3, 37]; when comparing CMT and no CMT groups the CMT might be linked to increased overall symptom load. Previous research has suggested a dose-response relationship between CMT and psychosis, where more severe CMT is associated with severity of psychotic symptoms [3, 23, 24]. Our findings provide additional support for such an association between CMT and total and negative symptom severity. Though causality between CMT and psychosis symptom severity cannot be established based on the current study, CMT be a risk factor for psychosis. This is supported by research showing that a reverse association is unlikely [38].

Regarding CMT and increased severity of negative symptoms in SSDs, the association is not yet well established [39]. In our findings, higher levels of CMT predicted more severe negative symptoms. Negative symptoms, as with CMT, are associated with worse treatment outcomes and less response to medical treatment [19, 20, 40, 41]. Consequently, negative symptoms are commonly found to be more difficult to treat and have a tendency to persist longer [42]. Thus, a better understanding of predictors of negative symptoms has both scientific and clinical value.

A possible moderating effect of having both parents with mental health problems was found for the relationship between overall CMT and the PANSS general psychopathology subscale. A measure of general psychopathology provides information of more global symptoms that could impact the overall symptom load, such as anxiety, depression and poor attention [31]. Research has suggested that having parental mental health problems increases the risk for several mental health disorders [17, 43]. Possibly, having parents with mental health problems could increase the risk of more global symptoms of psychopathology not necessary directly associated with psychosis.

Further, the present findings suggest that the CMT group as compared to the no CMT group have a more severe clinical presentation with more patients diagnosed with schizophrenia, more patient reporting depressive symptoms in addition to the increased symptoms of psychosis. This is in line with other studies showing CMT to be associated with other mental health problems [15, 44, 45], even though there seems to be some graded specificity were psychosis patients have been shown to have more types of CMT, and a higher proportion reports CMT as compared to e.g. anxiety and depression [44] but not substance abuse [45]. In line with this, Baldwin et al. found a somewhat higher effect size (Cohen’s d) for psychosis versus depression (0.34 versus 0.22) examining the effect of CMT on mental health [15]. It has been suggested that psychosis patients with CMT constitutes a particular subgroup in psychosis with worse prognosis, emphasizing the need for targeted treatment of trauma in this group [46].

The present study has several limitations. The study is based on a sample from a naturalistic, cross-sectional, pragmatic study, which entails that we cannot ascertain neither causal direction nor the possibility of other confounding variables, but in return provides improved external validity and generalizability. The sample size is too small to examine subtypes of CMT with sufficient power, and there may by relationships in the data not detected due to the small sample size. Studies have shown differential effect of types of CMT on mental health outcomes, e.g. emotional abuse may have a particularly stronger association to mental health [15]. This could not be tested in our study.

Further, the assessment of parental MHP was based on the evaluation by the included patients, not diagnosis from the parents’ medical records. It may be argued, however, that a child’s subjective experience of the parents’ mental health is the indication of mental health problem strong enough to warrants a child’s attention. The subjective experience could therefore be the most robust and valid variable, decreasing the chance of false positives and being more strongly related to the psychological distress CMT could potentially cause. In addition, a systematic review and meta-analyses found that the causal relationship between CMT and mental health was not moderated by prospective versus retrospective, nor longitudinal versus cross-sectional assessments of CMT, suggesting robustness of these assessments [15]. Still, the data is limited by not including information on severity or functionality of the parents, reducing the understanding of the qualitative and quantitative impact and mechanism of the MHP to CMT.

In addition, the relationship between MHP, CMT and psychosis may be influenced by the level of MHP vulnerability and risk (see e.g. [47]). The overall model statistics indicated poor fit, possibly indicating the complexity of predicting psychosis symptoms and corresponding lack of relevant variables included in the models. Still, the CMT-psychosis findings are deemed valid since they are supported by the group comparisons for psychosis symptoms and other studies [3, 37, 48]. Finally, to truly understand the differential effect of environmental versus genetic factors, genetically informative designs should be used in future studies (see e.g. [12]).

## Conclusion

In conclusion, this is one of few studies examining the role of parental mental health on the relationship between CMT and SSDs. The findings do not suggest that parental mental illness is a significantly moderating factor, but rather provide further support for an independent, dose-response relationship between CMT and SSDs. As such, effort might be advised to be put in the prevention, intervention and treatment of CMT in relation to psychosis development and prognosis.

## Supplementary Information

Supplementary Material 1.

## Data Availability

Data availability statement: According to Norwegian law, data sharing requires approvals from the Regional Committees for Medical and Health Research Ethics, and from the Data Protection Officer at Haukeland University Hospital, on the basis of specific research proposals. Inquiries regarding access to raw data should be made to Erik Johnsen (erik.johnsen@helse-bergen.no) as leader of the Bergen Psychosis Research Group.

## Abbreviations

CMT: Childhood maltreatment and trauma
SSDs: Schizophrenia spectrum disorders
MHP: Mental health problems
BeStInTro study: Bergen-Stavanger-Innsbruck-Trondheim study
CTQ: SF-Childhood Trauma Questionnaire-Short Form
DSM IV: Diagnostic and Statistical Manual of Mental Disorders, Fourth Edition
PANSS: Positive and Negative Syndrome Scale
SCI: PANSS-Structural Clinical Interview for the Positive and Negative Syndrome Scale
CDSS: Calgary Depression Scale for Schizophrenia
n: Number
M: Mean
SD: Standard deviations
Adj: Adjusted
REC West: Regional Committee for Medical and Health Research Ethics in Western Norway
DDD: Defined daily dose
DUP: Duration of untreated psychosis
CAUS: Clinical Alcohol Use Scale
CDUS: Clinical Drug Use Scale
CGI: Clinical Global Impression Scale
GAF: Global Assessment of Functioning
BMI: Body mass index
