# Supplementary material for: The relationship between childhood maltreatment and trauma and psychosis is not moderated by parental mental health
Source: BMC Psychiatry. 2025 Aug 6;25:766. doi: 10.1186/s12888-025-07190-8 (PMC12326774; doi:10.1186/s12888-025-07190-8)
Supplement: Supplementary file 1 — Supplementary Material 1. [file 12888_2025_7190_MOESM1_ESM.docx]

**Appendix A. Supplementary tables**

Tables (4 – 7) showing the full results from the first multiple regression analyses of CTQ-SF sum score and parental mental health problems as an interaction term on PANSS total and subscale scores, controlling for age and sex, including *t*-value and standard error (SE).

**Table 4**

Results of Multiple Regression Analyses of CTQ-SF Sum Score and Parental Mental Health Problems as an Interaction Term on PANSS Total Scale Score, Controlling for Age and Sex.

|  | PANSS total scale | | | |
| --- | --- | --- | --- | --- |
|  | Estimate^b^ | SE | *t* | *p* |
| Intercept^a^ | 70.616 | 6.147 | 11.488 | 0 |
| CMT^c^ | 0.222 | 0.112 | 1.990 | .049* |
| Age | - 0.215 | 0.126 | - 1.701 | .091* |
| Sex | - 1.081 | 3.206 | - 0.337 | .736 |
| Parental Mental Health Problems |  |  |  |  |
| Maternal | - 2.100 | 13.569 | - 0.155 | .877 |
| Paternal | 19.615 | 13.309 | 1.474 | .143 |
| Both | - 44.604 | 30.098 | - 1.482 | .141 |
| CMT X maternal | 0.112 | 0.300 | 0.373 | .710 |
| CMT X paternal | - 0.252 | 0.274 | - 0.920 | .360 |
| CMT X both | 0.852 | 0.668 | 1.275 | .205 |

Note. **p* <.05. *p* = p-value; *t* = *t*-value; SE = Standard error; PANSS = the Positive and Negative Syndrome Scale; CMT = Childhood maltreatment and trauma. CTQ-SF = Childhood Trauma Questionnaire Short-Form. ^a^ Mean value of dependent variables when all independent variables equal 0. ^b^Estimate of the expected change in independent variable with one unit change of dependent variable (*β)*. ^c^CTQ-SF sum score.

**Table 5**

Results of Multiple Regression Analyses of CTQ-SF Sum Score and Parental Mental Health Problems as an Interaction Term on PANSS Positive Subscale Score, Controlling for Age and Sex.

|  | PANSS positive subscale | | | |
| --- | --- | --- | --- | --- |
|  | Estimate^b^ | SE | *t* | *p* |
| Intercept^a^ | 17.898 | 2.071 | 8.642 | 0 |
| CMT^c^ | 0.046 | 0.037 | 1.252 | .213 |
| Age | 0.013 | 0.042 | 0.300 | .765 |
| Sex | - 0.997 | 1.076 | - 0.926 | .356 |
| Parental mental health problems |  |  |  |  |
| Maternal | - 1.171 | 4.574 | - 0.256 | .798 |
| Paternal | 0.285 | 4.486 | 0.064 | .949 |
| Both | - 10.199 | 10.152 | - 1.005 | .317 |
| CMT X maternal | 0.001 | 0.101 | 0.006 | .995 |
| CMT X paternal | - 0.020 | 0.092 | - 0.216 | .829 |
| CMT X both | 0.186 | 0.225 | 0.824 | .412 |

Note. **p* <.05. ***p* <.01. *p* = *p*-value; *t* = *t*-value; SE = Standard error; PANSS = the Positive and Negative Syndrome Scale; CMT = Childhood maltreatment and trauma. CTQ-SF = Childhood Trauma Questionnaire Short-Form. ^a^Mean value of dependent variables when all independent variables equal 0. ^b^Estimate of the expected change in independent variable with one unit change of dependent variable (*β)*. ^c^CTQ-SF sum score.

**Table 6**

Results of Multiple Regression Analyses of CTQ-SF Sum Score and Parental Mental Problems as an Interaction Term on PANSS Negative Subscale Score, Controlling for Age and Sex.

|  | PANSS negative subscale | | | |
| --- | --- | --- | --- | --- |
|  | Estimate^b^ | SE | *t* | *p* |
| Intercept^a^ | 16.836 | 2.100 | 8.019 | 0 |
| CMT^c^ | 0.087 | 0.038 | 2.280 | .024* |
| Age | - 0.134 | 0.043 | - 3.106 | .002** |
| Sex | 0.308 | 1.095 | 0.281 | .779 |
| Parental mental health problems |  |  |  |  |
| Maternal | 0.712 | 4.635 | 0.154 | .878 |
| Paternal | 8.661 | 4.546 | 1.905 | .059 |
| Both | - 2.665 | 10.281 | - 0.259 | .796 |
| CMT X maternal | 0.015 | 0.103 | 0.143 | .887 |
| CMT X paternal | - 0.121 | 0.094 | - 1.294 | .198 |
| CMT X both | - 0.023 | 0.228 | - 0.101 | .920 |

Note. **p* <.05. ***p* <.01. *p* = *p*-value; *t* = *t*-value; SE = Standard error; PANSS = the Positive and Negative Syndrome Scale; CMT = Childhood maltreatment and trauma. CTQ-SF = Childhood Trauma Questionnaire Short-Form. ^a^Mean value of dependent variables when all independent variables equal 0. ^b^Estimate of the expected change in independent variable with one unit change of dependent variable (*β)*. ^c^CTQ-SF sum score.

**Table 7**

Results of Multiple Regression Analyses of CTQ-SF Sum Score and Parental Mental Disorders as an Interaction Term on PANSS General Psychopathology Subscale Score. Controlling for Age and Sex.

|  | PANSS general psychopathology subscale | | | |
| --- | --- | --- | --- | --- |
|  | Estimate^b^ | SE | *t* | *p* |
| Intercept^a^ | 35.963 | 3.197 | 11.250 | 0 |
| CMT^c^ | 0.084 | 0.057 | 1.462 | .146 |
| Age | - 0.091 | 0.066 | - 1.391 | .167 |
| Sex | - 0.312 | 1.661 | - 0.188 | .851 |
| Parental Mental Health Problems |  |  |  |  |
| Maternal | - 1.782 | 7.061 | - 0.252 | .801 |
| Paternal | 10.523 | 6.925 | 1.520 | .131 |
| Both | - 31.910 | 15.671 | - 2.036 | .044* |
| CMT X maternal | 0.101 | 0.156 | 0.647 | .519 |
| CMT X paternal | - 0.107 | 0.142 | - 0.750 | .455 |
| CMT X both | 0.695 | 0.348 | 1.999 | .048* |

Note. **p* <.05. ***p* <.01. *p* = *p*-value; *t* = *t*-value; SE = Standard error; PANSS = the Positive and Negative Syndrome Scale; CMT = Childhood maltreatment and trauma. CTQ-SF = Childhood Trauma Questionnaire Short-Form. ^a^Mean value of dependent variables when all independent variables equal 0. ^b^Estimate of the expected change in independent variable with one unit change of dependent variable (*β)*. ^c^CTQ-SF sum score.
